# Genetic Testing in Patients with Autoimmune Lymphoproliferative Syndrome: Experience of 802 Patients at Cincinnati Children’s Hospital Medical Center

**DOI:** 10.1007/s10875-024-01772-z

**Published:** 2024-07-26

**Authors:** Xinxiu Xu, James Denton, Yaning Wu, Jie Liu, Qiaoning Guan, D. Brian Dawson, Jack Bleesing, Wenying Zhang

**Affiliations:** 1 Division of Human Genetics, Cincinnati Children’s Hospital Medical Center, Cincinnati, OH USA; 2 Department of Pediatrics, University of Cincinnati College of Medicine, Cincinnati, OH USA; 3 Division of Bone Marrow Transplantation and Immune Deficiency, Cincinnati Children’s Hospital Medical Center, Cincinnati, OH USA

**Keywords:** ALPS, Autoimmune lymphoproliferative syndrome, Immunodeficiency, Next generation sequencing, Genetics, *FAS*

## Abstract

Autoimmune lymphoproliferative syndrome (ALPS) is a rare genetic disorder featuring chronic lymphadenopathy, splenomegaly, cytopenias, and increased lymphoma risk. Differentiating ALPS from immunodeficiencies with overlapping symptoms is challenging. This study evaluated the performance and the diagnostic yield of a 15-gene NGS panel for ALPS at Cincinnati Children’s Hospital Medical Center. Samples from 802 patients submitted for ALPS NGS panel were studied between May 2014 and January 2023. A total of 62 patients (7.7%) had a definite diagnosis: 52/62 cases (84%) showed 37 unique pathogenic/likely pathogenic germline *FAS* variants supporting ALPS diagnosis (6.5%, 52/802). The ALPS diagnostic yield increased to 30% in patients who additionally fulfilled abnormal ALPS immunology findings criteria. 17/37 (46%) diagnostic *FAS* variants were novel variants reported for the first time in ALPS. 10/802 cases (1.2%) showed diagnostic findings in five genes (*ADA2*,* CTLA4*,* KRAS*,* MAGT1*,* NRAS*) which are related to autoimmune lymphoproliferative immunodeficiency (ALPID). Family studies enabled the reclassification of variants of unknown significance (VUS) and also the identification of at-risk family members of *FAS*-positive patients, which helped in the follow-up diagnosis and treatment. Alongside family studies, complete clinical phenotypes and abnormal ALPS immunology and Fas-mediated apoptosis results helped clarify uncertain genetic findings. This study describes the largest cohort of genetic testing for suspected ALPS in North America and highlights the effectiveness of the ALPS NGS panel in distinguishing ALPS from non-ALPS immunodeficiencies. More comprehensive assessment from exome or genome sequencing could be considered for undefined ALPS-U patients or non-ALPS immunodeficiencies after weighing cost, completeness, and timeliness of different genetic testing options.

**Supplementary Information:**

The online version contains supplementary material available at 10.1007/s10875-024-01772-z.

## Introduction

Autoimmune lymphoproliferative syndrome (ALPS) is a rare primary immunodeficiency disorder of defective Fas-mediated apoptosis (restimulation-induced cell death), featuring chronic lymphadenopathy, splenomegaly, cytopenias, and increased lymphoma risk. While typically seen in childhood, symptoms can manifest at any age. The diagnostic approach to suspicious patients tends to be broad and often includes imaging studies as well as invasive diagnostic procedures, such as the biopsy of lymph nodes and/or other tissues. The typical biomarker for ALPS is an increased number of a normally uncommon population of “double-negative TCRαβ + CD3 + CD4- CD8- T cells” (DNTs); other characteristic laboratory abnormalities include defective lymphocyte apoptosis, elevated levels of interleukin (IL) IL-10, IL-18, vitamin B12, and soluble FAS-ligand (sFASL) [1].

The molecular basis for ALPS was gradually deciphered with the advances in sequencing and molecular technology. In 1995, heterozygous pathogenic variants in *FAS* were first reported in children with ALPS [2, 3]. A year later, Wu et al. reported a heterozygous 84-bp deletion in exon 4 of *FASLG* in a patient originally thought to have systemic lupus erythematosus (SLE) [4], but later was considered more consistent with ALPS type Ib [5]. Later publications showed monoallelic loss-of-function variants in *FASLG* are tolerated. ALPS-FASLG (ALPS type Ib) is likely caused by biallelic loss-of-function variants [6]. In 1999, Wang et al. identified heterozygous pathogenic variants in *CASP10* in ALPS type II [5], but its pathogenicity has been debated [7]. In 2004, somatic pathogenic *FAS* variants were reported in ALPS [8]. In 2009, a genetic diagnosis was recommended to be part of the ALPS diagnostic criteria and the ALPS classification was recommended to be based on genes with pathogenic variants detected [9]. Recently, germline heterozygous *FADD* mutations, coupled with a somatic loss of heterozygosity (LOH) in DNTs, have been reported to cause ALPS [10] and was classified as ALPS-FADD [7]. Whereas the biallelic mutations in *FADD* are associated with immunodeficiency 90 with encephalopathy, functional hyposplenia, and hepatic dysfunction (Online Mendelian Inheritance in Man (OMIM) #613759), which is not an ALPS variant. It has been recently proposed to restrict the ALPS classification to conditions with clear evidence of FAS signaling defect and resulting ALPS specific clinical and laboratory manifestations [7]. Autoimmune lymphoproliferative immunodeficiency (ALPID) was proposed as a clinical “umbrella” term that includes conditions that may initially present with an ALPS-like phenotype and biomarkers at the borders, but show additional non-ALPS symptoms later on [7].

With more ALPS patients having genetic defects identified and reported, we developed a clinical *FAS* gene sequence test in 2005, then *FASLG* and *CASP10* sequencing tests in 2009. With the advance of the next-generation sequencing (NGS) technology, in 2014, we developed an NGS panel that includes *FAS*,* FASLG*,* FADD* (monoallelic mutations), and *CASP10*, as well as genes associated with top differential diagnoses of ALPID, including *CASP8*,* CTLA4*, *FADD* (biallelic mutations), *ITK*,* MAGT1*,* PRKCD*,* STAT3*, *KRAS*, *LRBA*, *NRAS*, *RASGRP1*, and *ADA2 (CECR1)*.

In this paper, we present a retrospective study of 802 patients undergoing the ALPS NGS panel at Cincinnati Children’s Hospital Medical Center (CCHMC). We report on the primary molecular and immune characteristics of ALPS and some ALPID and the difficulties encountered during genetic testing. Furthermore, we assess and summarize the clinical utility of our ALPS testing algorithm (Suppl. Figure 1), which aligns with the testing guideline outlined in the 2009 NIH international workshop for the genetic diagnosis of ALPS patients [9].

## Methods

### Patient’s Enrollment

A retrospective study of 802 individuals who had an ALPS NGS panel performed at CCHMC from May 2014 to January 2023 was conducted to evaluate the performance of the test. The patient’s clinical phenotypes based on review of the requisitions and/or chart review, the pathogenicity of variants, and, where available, the laboratory findings of the ALPS Immune-panel and Fas-mediated lymphocyte apoptosis were evaluated and summarized (Table 1). Testing was ordered by clinicians from various institutions.

**Table 1:** The demographic and result overview of the 802-patient cohort with suspected ALPS

Characteristic		Number	%
Sex			
	Male	504	62.8
	Female	294	36.7
	Unknown	4	0.5
Age at genetic test (years)			
	Median = 12.07 (Range 0-75.8)		
	0–1	23	2.9
	1–12	375	46.8
	12–19	291	36.3
	>=20	113	14.1
Race/Ethnicity*			
	European American	219	40.0
	African American	62	11.3
	Middle Eastern	49	8.9
	Other	46	8.4
	Asian	12	2.2
	Hispanic	10	1.8
	European American/African American	6	1.1
	Native American or Alaskan	2	0.4
	Unknown	142	25.9
Genetic test result			
	Positive	62	7.7
	Inconclusive	66	8.2
	Negative	674	84.0
Family Study**			
	Process family study	23	18.0
ALPS Immue-panel Test***			
	Consistent	7	2.8
	Suspicious	13	5.1
	Negative	81	31.9
	Not tested	153	60.2
Fas-mediated Apoptosis test***			
	Decreased	7	2.8
	Negative	16	6.3
	Not tested	231	90.9

* 548 patients from May 2014 to October 2019 were collected with race/ethnicity information

** 128 patients with VUS, likely pathogenic and pathogenic findings in NGS testing

*** 254 patients from November 2019 to January 2023 were systematically reviewed for the ALPS Immune-panel test, the Fas-mediated apoptosis test, and the ALPS NGS test results

### DNA Extraction and NGS test

Genomic DNA was extracted from peripheral blood. NGS was conducted on 9 genes (*CASP8*, *CASP10*, *FADD*, *FAS*, *FASLG*, *ITK*, *KRAS*, *MAGT1*, *NRAS*) for 548 cases from May 2014 to October 2019 or 15 genes (additional genes: *ADA2*, *CTLA4*, *LRBA*, *PRKCD*, *RASGRP1*, and *STAT3*) for 254 cases from November 2019 to January 2023. Variants were classified according to the ACMG guidelines [11]. Somatic variants with > = 5% allele fraction in *FAS* were targeted for cases performed after November 2019. Supplementary material.

### Flow Cytometry ALPS Immune-Panel

An ALPS Immune-panel was completed for 101 of 254 patients tested for ALPS NGS panel from November 2019 to January 2023. Due to the change of the institutional LIMS system, we had limited access to the ALPS Immune-panel results completed before November 2019. Therefore, we only reviewed the ALPS Immune-panel results for patients with abnormal ALPS NGS panel (30 patients).

This flow cytometry panel interrogates 4 parameters suggestive of ALPS (TCRαβ DNTCs > 2% or > 68 cells/ul; B220 + TCRαβ DNTCs > 60%; CD3 + CD25+ / HLA DR ratio < 1.0; CD27 + B cells < 15%), based on published results [12–14]. A score system was utilized with 4/4 as consistent with ALPS; 3/4 as suspicious for ALPS; 2/4 as not typical for ALPS; and 1/4 or 0/4 as not consistent for ALPS. Peripheral lymphocyte subsets were evaluated from whole blood using an 8-color immunostaining panel (lyse and wash procedure), a FACS Canto II flow cytometer (BD) equipped with three lasers (blue, red, violet), FACSDiva software (BD), and a large panel of RUO mAbs and fluorochromes variously combined (all BD). This panel is clinically available (https://www.testmenu.com/cincinnatichildrens/Tests/660966).

### Fas-mediated Apoptosis Test

An in vitro Fas-mediated apoptosis test was conducted on 23 out of 254 patients tested for ALPS NGS panel from November 2019 to January 2023. Due to the Institutional LIMS change, we only reviewed the apoptosis results for patients with abnormal ALPS NGS panel (11 patients) for cases completed before November 2019.

In short, peripheral blood mononuclear cells were suspended in complete RPMI 1640 medium and T cells were activated with Concanavalin A. Activation was assessed using CD3, CD8 and CD25. After 4 days, activated T cells were washed and then cultured in medium supplemented with recombinant human IL-2 for 7 days. Expanded T cells were then plated in duplicate in 96-well plates and treated with agonistic anti-Fas antibody (APO-1-3, Alexis Biochemicals) and Protein A in the presence of IL-2 to evaluate Fas-mediated lymphocyte apoptosis. Twenty-one hours after treatment, cells were stained with propidium iodide and analyzed by flow cytometry. Cell death was quantified as % cell loss = (1-(% viable cells, treated / % viable cells, untreated)) x 100. Percent Cell Loss was reported from a 500 ng/mL concentration of APO-1-3 and Protein A. Fas function was defined as defective when cell survival was out of the reference range (68–93%). This test is clinically available (https://www.testmenu.com/cincinnatichildrens/Tests/660968).

## Results

### Demographic Findings in 802 Patients with Suspected ALPS

Between May 2014 and January 2023, 802 patients with suspected ALPS from North America were referred from different institutions and underwent ALPS NGS testing. The characteristics of the cohort are summarized in Tables 1; 63% (504/802) were male and 37% (294/802) were female. The median age at testing was 12 years (ranging from 1 month to 76 years). Of the 548 patients tested from May 2014 to October 2019 with race/ethnicity information, almost 40% (219/548) were European American.

### The Diagnostic Yield of ALPS NGS Panel in 802 Patients

The diagnostic yield of ALPS NGS panel in 802 patients with suspected ALPS was 7.7% (62/802). Of the 62 positive cases, 52/62 (84%) had *FAS* pathogenic/likely pathogenic variants supporting the diagnosis of ALPS. Of the 52 *FAS* positive cases, 73% (38/52) were male and 27% (14/52) were female, with a male predominance as previously suggested [15].

**Fig. 1:**
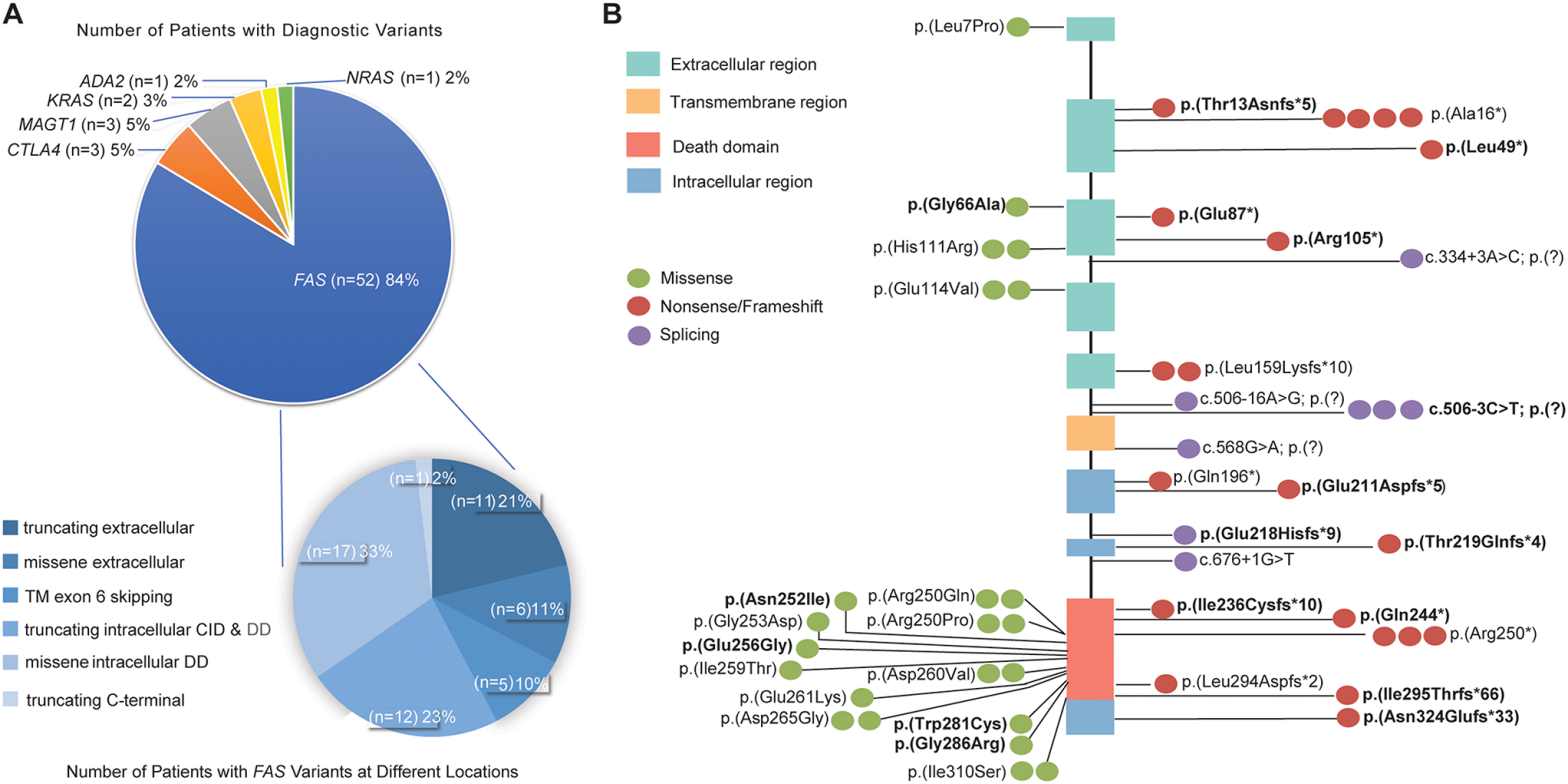
(**A**) Pie charts reporting the frequency of diagnostic variants identified in the 802-patient cohort, grouped by gene, and the frequency of *FAS* variants grouped by domain. (**B**) Schematic diagram of the locations of 37 diagnosis germline variants in 52 patients with respect to the predicted domains of *FAS*. Each dot represents one patient and novel variants are bolded. The intracellular death domain (AA230-314 region) is highlighted in orange-red color. CID, Calcium Inducing Domain; DD, Death Doman

The remaining 10/62 (16%) had pathogenic/likely pathogenic variants in *CLTA4*,* MAGT1*,* KRAS*,* ADA2*, and *NRAS* (Fig. 1A).

No diagnostic variants were detected in *FASLG* and *CASP10*, suggesting ALPS-FASLG and ALPS-CASP10 are rare.

66 cases had variants of unknown significance (VUS).

Among 62 positive cases and 66 cases with VUS (total 128 cases), 23 cases (18%) underwent follow-up family studies in our institution (Table 1). All 23 families were tested for the variants in *FAS*. Some representative family study results were shown in Fig. 3.

### Genetic Findings in *FAS*

52 out of 802 (6.5%) patients had a heterozygous pathogenic or likely pathogenic variant in *FAS*. 37 unique *FAS* variants were detected in 52 cases, with 11 (30%) variants shared by multiple patients (Fig. 1B; Table 2). 20/37 (54%) variants have been previously reported in patients with ALPS. 17/37 (46%) variants were novel variants which had not been previously reported in any ALPS patients (bold in Fig. 1B; Table 2). Of these 52 cases, 22 (42%) were heterozygous for a truncating variant or a missense variant in the extracellular domain (ECD) or a variant resulting in the transmembrane (TM) domain-coding exon 6 skipping. The effect of these variants was reported to be either decreased FAS expression or localization, or inability to bind FAS ligand with reduced FAS ligand-induced apoptosis [16]. 30/52 (58%) cases were heterozygous for variants in the intracellular domain (ICD), of which, 17 (57%) were with missense variants in the death domain, which plays a key role in the death-inducing signaling [16] (Fig. 1B; Table 2). Most of the 17 novel variants were truncating variants or missense variants in the death domain, except c.197G>C p.(Gly66Ala). This variant is in ECD and has not been reported in the literature, but a different variant at the same nucleotide position resulting in a different amino acid change, c.197G>A p.(Gly66Asp), has been reported in a patient with ALPS [17], suggesting the functional importance of this locus. p.(Gly66Ala) is predicted to be deleterious (REVEL score of 0.854) and was classified as likely pathogenic based on the ACMG variant classification criteria [11].

**Table 2:** Diagnostic pathogenic or likely pathogenic variants in the *FAS* gene in 802 patients with suspected ALPS

Pt ID	Age (yrs)	Sex	Race/ ethnicity	Variant	Variant type	Classifi-cation	In silico	ClinVar	FAS domain	ALPS Immune-panel	Clinical indication		Ref
											ALPS specific	Non-ALPS specific	
1	14	F	Unknown	c.20T>C; p.(Leu7Pro)	missense	LP	deleterious effect	LP(2), 1213255	extracellular	N/A	anemia, neutropenia, thrombocytopenia hepatosplenomegaly, a history of recurrent infections	eczema, dermatitis, and small lymph nodes	[16]
2	16	M	Other	c.37dupA; p.(Thr13Asnfs*5)	frameshift	Path	predicted null variant	not reported	extracellular	*not consistent with a diagnosis of ALPS (twice)	N/A	N/A	This study
3	13	M	Unknown	c.46_47del; p.(Ala16*)	nonsense	path	predicted null variant	Path(2), 872242	extracellular	consistent with ALPS	clinical diagnosis of ALPS, with recurrent submandibular/auricular lymphadenopathy and abnormal T cell function	patellofemoral pain syndrome	[16, 45]
4	16	M	European American	c.46_47del; p.(Ala16*)	nonsense	Path	predicted null variant	Path(2), 872242	extracellular	*not typical for a diagnosis of ALPS	hepatosplenomegaly, a mediastinal mass, enlarged lymph nodes, cytopenias, anemia, leukopenia & thrombocytopenia	follicular hyperplasia	[16, 45]
5	6	M	Unknown	c.46_47del; p.(Ala16*)	nonsense	Path	predicted null variant	Path(2), 872242	extracellular	N/A	hepatosplenomegaly enlarged lymph nodes, anemia, thrombocytopenia, reported abnormal ALPS panel, and autoimmune cytopenias	N/A	[16, 45]
6	4	F	European American	c.46_47del; p.(Ala16*)	nonsense	Path	predicted null variant	Path(2), 872242	extracellular	*not consistent with a diagnosis of ALPS	hepatosplenomegaly, anemia, neutropenia/ leukopenia, thrombocytopenia and abnormal lymphocyte subsets	N/A	[16, 45]
7	13	M	European American	c.146T>A; p.(Leu49*)	nonsense	Path	predicted null variant	Not reported	extracellular	consistent with ALPS	N/A	N/A	This study
8	50	M	European American	**c.197G>C; p.(Gly66Ala)**	missense	LP	deleterious effect	not reported	extracellular	N/A	hepatosplenomegaly and enlarged lymph nodes	N/A	This study
9	3	M	European American	c.259G>T; p.(Glu87*)	nonsense	Path	predicted null variant	Path(1). 1451181	extracellular	suspicious for ALPS (1st time); *Not typical for a diagnosis of ALPS.(6 mo later)	NA	N/A	This study
10	2	F	Other	c.312dup; p.(Arg105*)	nonsense	Path	predicted null variant	not reported	extracellular	suspicious for ALPS	hepatosplenomegaly, leukopenia, neutropenia, and red cell anemia	N/A	This study
11	3	M	European American	c.332A>G; p.(His111Arg)	missense	Path	uncertain impact	Path(1). 1395111	extracellular	consistent with ALPS	enlarged lymph nodes, red cell anemia, reported abnormal ALPS panel	increased IgG and IgA	[16, 45]
12	12	M	European American	c.332A>G; p.(His111Arg)	missense	Path	uncertain impact	Path(1), 1395111	extracellular	consistent with ALPS	splenomegaly, enlarged lymph nodes, and cytopenias	N/A	[16, 45]
13	11	F	Unknown	c.334+3A>C; p.(?)	splicing	LP	may disrupt a nearby splice donor	VUS(1), 1406327	extracellular	suspicious for ALPS	N/A	N/A	[31]
14	24	M	African American	c.341A>T; p.(Glu114Val)	missense	LP	deleterious effect	not reported	extracellular	N/A	N/A	N/A	[16]
15	7	M	African American	c.341A>T; p.(Glu114Val)	missense	LP	deleterious effect	not reported	extracellular	N/A	N/A	N/A	[16]
16	32	F	European American	c.475_489delinsA; p.(Leu159Lysfs*10)	frameshift	path	Predicted null variant	not reported	extracellular	*not consistent with a diagnosis of ALPS	Positive FH, son with ALPS	N/A	[16, 46]
17	2	M	European American	c.475_489delinsA; p.(Leu159Lysfs*10)	frameshift	Path	predicted null variant	not reported	extracellular	suspicious for ALPS	N/A	N/A	[16, 46]
18	15	M	European American	c.506-16A>G; p.(?)	splicing	LP	uncertain impact	not reported	transmembrane	N/A	hepatosplenomegaly, enlarged lymph nodes, lymphoma, and PTLD with > 25% DNT cells	a clinical history of cardiac transplant, sensorineural deafness	[16, 33, 47]
19	15	M	European American	**c.506-3C>T; p.(?)**	splicing	LP	uncertain impact	not reported	transmembrane	N/A	hepatosplenomegaly, enlarged lymph nodes, red cell anemia, thrombocytopenia, and reported abnormal ALPS panel	N/A	This study [30]
20	12	M	Other	**c.506-3C>T; p.(?)**	splicing	LP	uncertain impact	not reported	transmembrane	N/A	enlarged lymph nodes, red cell anemia, and thrombocytopenia	N/A	This study [29, 30]
21	5	M	European American	**c.506-3C>T; p.(?)**	splicing	LP	uncertain impact	not reported	transmembrane	*not typical for a diagnosis of ALPS	leukopenia/neutropenia, red cell anemia, thrombocytopenia, splenomegaly, paternal family history of ALPS	epistaxis, intermittent rashes of ankles, small platelets	This study [29, 30]
22	0.9	M	European American	c.568G>A; p.(?)	splicing	LP	predicted null variant	not reported	transmembrane	N/A	N/A	fever and rash/dermatitis	[16, 33] AKA c.538 C > T
23	5	M	European American	c.586C>T; p.(Gln196*)	nonsense	Path	predicted null variant	not reported	intracellular	consistent with ALPS	(hepato)splenomegaly, enlarged lymph nodes, leukopenia/neutropenia, red cell anemia, and thrombocytopenia	fevers	[48]
24	21	M	Unknown	c.633del; p.(Glu211Aspfs*5)	frameshift	path	predicted null variant	not reported	intracellular	suspicious for ALPS	N/A	N/A	This study
25	6	F	European American	c.652-7_656delinsCATTTT; p.(Glu218Hisfs*9)	frameshift	LP	predicted null variant	not reported	intracellular	N/A	(hepato)splenomegaly, enlarged lymph nodes, and abnormal lymphocyte subsets	N/A	This study
26	0	M	Asian American	c.655del; p.(Thr219Glnfs*4)	frameshift	LP	predicted null variant	not reported	intracellular	N/A	hepatosplenomegaly, anemia, recurrent viral infection, thrombocytopenia	rash and dermatitis	This study
27	35	F	Latino-Hispanic	c.676+1G>T; p.(?)	splicing	Path	predicted null variant	Path(1), 1070181	intracellular	N/A	enlarged lymph nodes, follicular helper T-cell related lymphoma, and iron deficiency anemia	N/A	[16, 49–51]
28	1	M	Native American or Alaskan	c.706_707del; p.(Ile236Cysfs*10)	frameshift	Path	predicted null variant	Not reported	Intracellular (death domain)	N/A	hepato/splenomegaly, lymphadenopathy, cytopenias, reported abnormal ALPS panel, elevated DNTCs	failure to thrive, dysmorphic facies, small platelets	This study
29	16	M	Unknown	c.730C>T; p.(Gln244*)	nonsense	Path	predicted null variant	Not report	Intracellular (death domain)	consistent with ALPS	N/A	N/A	This study
30	4	F	Unknown	c.748C>T; p.(Arg250*)	nonsense	Path	predicted null variant	Path(4)/LP(2), 802620	Intracellular (death domain)	N/A	(hepato)splenomegaly, recurrent viral infections, and increased DNTCs	N/A	[31, 45, 52]
31	16	M	Unknown	c.748C>T; p.(Arg250*)	nonsense	Path	predicted null variant	Path(4)/LP(2), 802620	Intracellular (death domain)	suspicious for ALPS	hepatosplenomegaly, reported abnormal ALPS panel, thrombocytopenia, family history of lymphoma and myeloproliferative neoplasm	N/A	[31, 45, 52]
32	29	M	European American	c.748C>T; p.(Arg250*)	nonsense	Path	predicted null variant	Path(4)/LP(2), 802620	Intracellular (death domain)	N/A	chronic non-malignant, non-infectious lymphadenopathy, splenomegaly, defective lymphocytes apoptosis and elevated DNTCs	N/A	[31, 45, 52]
33	21	M	European American	c.749G>A; p.(Arg250Gln)	missense	Path	deleterious effect	Path(3)/LP(1), 1070182	Intracellular (death domain)	consistent with ALPS	hepatosplenomegaly, enlarged lymph nodes, Hodgkin lymphoma which is in remission, and thrombocytopenia	fevers	[16, 28, 31, 45, 53]
34	12	M	African American	c.749G>A; p.(Arg250Gln)	missense	Path	deleterious effect	Path(3)/LP(1), 1070182	Intracellular (death domain)	N/A	symptoms consistent with ALPS per report	N/A	[16, 28, 31, 45, 53]
35	2	M	European American	c.749G>C; p.(Arg250Pro)	missense	Path	Uncertain impact	Path(4), 16505	Intracellular (death domain)	N/A	hepatosplenomegaly, enlarged lymph nodes, abnormal lymphocyte subsets	N/A	[28, 45, 54]
36	26	M	European American	c.749G>C; p.(Arg250Pro)	missense	Path	Uncertain impact	Path(4), 16505	Intracellular (death domain)	suspicious for ALPS	(hepato)splenomegaly, enlarged lymph nodes, and abnormal lymphocyte subsets	N/A	[28, 45, 54]
37	17	M	European American	**c.755A>T; p.(Asn252Ile)**	missense	LP	deleterious effect	not reported	Intracellular (death domain)	suspicious for ALPS (twice)	hepatosplenomegaly, thrombocytopenia and reported abnormal ALPS panel	N/A	This study
38	11	M	Polish	c.758G>A; p.(Gly253Asp)	missense	LP	uncertain impact	Not reported	Intracellular (death domain)	N/A	hepatosplenomegaly, enlarged lymph nodes, anaplastic large cell lymphoma, thrombocytopenia	rash/dermatitis	[51, 55]
39	2	M	European American/African American	**c.767A>G;p.(Glu256Gly)**	missense	LP	deleterious effect	VUS(4), 1398630	Intracellular (death domain)	consistent with ALPS	N/A	N/A	This study
40	1	M	Latino-Hispanic	c.776T>C; p.(Ile259Thr)	missense	LP	uncertain impact	VUS(1), 860668	Intracellular (death domain)	N/A	chronic/recurrent lymphadenopathy, [hepato]splenomegaly, and autoimmune disease affecting blood cells and other tissues	N/A	[56]
41	58	F	European American	c.779A>T; p.(Asp260Val)	missense	path	uncertain impact	Path(2), 16504	Intracellular (death domain)	suspicious for ALPS (twice)	suspected ALPS per report	N/A	[25, 57]
42	0	F	European American	c.779A>T; p.(Asp260Val)	missense	Path	uncertain impact	Path(2), 16504	Intracellular (death domain)	*not consistent with a diagnosis of ALPS	hepatosplenomegaly, cytopenias, and reported abnormal ALPS panel	N/A	[25, 57]
43	0	M	Unknown	c.781G>A; p.(Glu261Lys)	missense	LP	uncertain impact	VUS(1), 2125605	Intracellular (death domain)	consistent with ALPS	hepatosplenomegaly, inguinal lymphadenopathy, enlarged lymph nodes in abdominal/pelvis region and history of anemia	N/A	[57–59]
44	6	F	Other	c.794A>G; p.(Asp265Gly)	missense	LP	deleterious effect	not reported	Intracellular (death domain)	consistent with ALPS	hepatosplenomegaly, enlarged lymph nodes, cytopenias	small platelets	[41, 42]
45	8	M	Unknown	c.794A>G; p.(Asp265Gly)	missense	LP	deleterious effect	not reported	Intracellular (death domain)	N/A	hepatosplenomegaly, enlarged lymph nodes and elevated DNTCs, maternal positive family history	N/A	[41, 42]
46	13	M	European American	**c.843G>T; p.(Trp281Cys)**	missense	LP	deleterious effect	not reported	Intracellular (death domain)	N/A	chronic submandibular lymphadenopathy, mild pancytopenia, splenomegaly, elevated DNTC’s, markedly elevated B12	N/A	This study
47	3	F	Unknown	**c.856G>A;p.(Gly286Arg)**	missense	LP	uncertain impact	not reported	Intracellular (death domain)	suspicious for ALPS.	N/A	N/A	This study
48	3	M	Unknown	c.879_880del; p.(Leu294Aspfs*2)	frameshift	Path	predicted null variant	Path(2), 265400	Intracellular (death domain)	Known ALPS patient (consistent with ALPS)	N/A	N/A	[45]
49	21	M	European American	c.884_890delinsCTAAGG; p.(Ile295Thrfs*66)	frameshift	Path	predicted null variant	Not reported	Intracellular (death domain)	N/A	clinical diagnosis of ALPS per report	N/A	This study
50	16	M	European American	c.929T>G; p.(Ile310Ser)	missense	LP	deleterious effect	VUS(1), 1406336	Intracellular (death domain)	N/A	hepato/splenomegaly, pancytopenia, reported abnormal ALPS panel	fevers, small platelet, BMF	[45]
51	12	F	Unknown	c.929T>G; p.(Ile310Ser)	missense	LP	deleterious effect	VUS(1), 1406336	Intracellular (death domain)	N/A	severe neutropenia, lymphadenopathy, splenomegaly, pancytopenia, positive direct Coombs test	antiphospholipid antibody positive, elevated IgE level, and high total IgG	[45]
52	9	F	Unknown	c.970_982del; p.(Asn324Glufs*33)	frameshift	LP	predicted null variant	not reported	intracellular	*not typical for a diagnosis of ALPS	hepatosplenomegaly, cytopenia, leukopenia, red cell anemia and neutropenia	N/A	This study

All *FAS* variants are heterozygous. All variants are absent from gnomAD except for Pts 14 and 15 (MAF is 0.003% in all populations and 0.01% in African American). Nine patients had the in-house Fas-mediated Apoptosis performed and with “decreased” results for Pts 2, 16, 31, 33, 44, 48 & 52 and “markedly decreased” results for Pts 41 and 43. Pts 16, 21, 31, 43 & 45 were reported to have a family history of ALPS. Bold means novel variants

Age: age of testing; FAS reference sequence: NM_000043.5; Pt = Patient; M = Male; F = Female; FH = Family history; Path = Pathogenic; LP = Likely Pathogenic; ALPS = Autoimmune Lymphoproliferative Syndrome; DNTC = Double Negative T Cell; AKA = also known as; PTLD = post-transplant lymphoproliferative disease

* Immunosuppressive therapy may affect the ALPS Immune-panel test

The ALPS NGS panel also tested for somatic *FAS* variants with allele fraction (AF) > = 5% for 254 cases processed from November 2019 to January 2023. However, no somatic *FAS* variants were detected.

### Genetic Findings in Other Genes Related to ALPID

Given the similar clinical features associated with ALPS and other lymphoproliferative disorders, our ALPS NGS panel also includes genes associated with the top differential diagnoses of ALPID. There were 10 cases that showed a diagnostic result in five genes associated with ALPID (Fig. 1A; Table 3), including one homozygous variant in *ADA2* that causes vasculitis, autoinflammation, immunodeficiency, and hematologic defects [18]; three heterozygous variants in *CTLA4* that cause immune dysregulation with autoimmunity, immunodeficiency, and lymphoproliferation [19, 20]; three likely heterozygous variants in *KRAS* or *NRAS* that cause RAS-associated autoimmune leukoproliferative disorder (RALD) [19, 21, 22]; and three hemizygous variants in *MAGT1* that causes immunodeficiency [23, 24]. Additionally, there were three cases with a heterozygous variant but an uncertain diagnostic result because the genes are associated with autosome recessive diseases and a second variant was not detected (Suppl. Table 1).

**Table 3:** Diagnostic pathogenic or likely pathogenic variants in other ALPID genes in 802 patients with suspected ALPS

Pt ID	Age (yrs)	Sex	Race/ ethnicity	Gene	OMIM	Variant	Variant type	Zygosity	Classification	In silico	gnomAD	ClinVar	ALPS Immune-panel	Fas-mediated apoptosis	Family history and clinical indication	Ref
53	13	M	other	*ADA2*	Vasculitis, autoinflammation, immunodeficiency, and hematologic defects syndrome (AR)	c.1072G>A; p.(Gly358Arg)	missense	HOMO	Path	deleterious effect	All: 0.002% SA: 0.01%	PA (2), Uncertain (1), 420337	N/A	N/A	positive FH, hepato/splenomegaly	[60–64]
54	9	F	Unknown	*CTLA4*	Immune dysregulation with autoimmunity, immunodeficiency, and lymphoproliferation (AD)	c.407C>T; p.(Pro136Leu)	missense	HET	LP	uncertain impact	Absent	LP(1),1,711,524	not consistent with a diagnosis of ALPS	N/A	NA	[65, 66]
55	5	F	African American	*CTLA4*	Immune dysregulation with autoimmunity, immunodeficiency, and lymphoproliferation (AD)	**c.458-1G>A; p.(?)**	splicing	HET	LP	predicted null variant	Absent	Not reported	not typical for a diagnosis of ALPS	N/A	hepato/splenomegaly, cytopenia, recurrent fever, lymphadenopathy, pulmonary infiltrates	This study
56	17	F	Latino-Hispanic	*CTLA4*	Immune dysregulation with autoimmunity, immunodeficiency, and lymphoproliferation (AD)	c.151C>T; p.(Arg51*)	stop gain	HET	Path	predicted null variant	Absent	Path(4), 161109	not consistent with a diagnosis of ALPS	N/A	(hepato)splenomegaly, lethargy, Joint pain, enlarged lymph nodes, alopecia, eczema, rash, leukopenia/ neutropenia, abnormal lymphocyte subsets, history of ITP	[19, 20, 67, 68]
57	18	F	Unknown	*KRAS*	RAS-associated autoimmune leukoproliferative disorder (AD)	c.37G>T; p.(Gly13Cys)	missense	HET	Path	deleterious effect	Absent	Path(7)/LP(1), 45123	not typical for diagnosis of ALPS	N/A	fever, splenomegaly, respiratory failure, pulmonary hypertension, recurrent infections, thrombocytopenia, abnormal lymphocyte subsets, low or absent NK function, elevated ferritin, elevated soluble IL2Ra, short stature, and delayed puberty	[69]
58	2	F	Other	*KRAS*	RAS-associated autoimmune leukoproliferative disorder (AD)	c.37G>T; p.(Gly13Cys)	missense	HET	Path	deleterious effect	Absent	Path(7)/LP(1), 45123	not consistent with a diagnosis of ALPS	N/A	fever, splenomegaly, pulmonary hypertension, thrombocytopenia, abnormal lymphocytes	[69]
59	9	M	African American	*MAGT1*	Immunodeficiency, X-linked, with magnesium defect, Epstein-Barr virus infection and neoplasia (XLR)	c.361_363delinsTATGCAG; p.(Val121Tyrfs*7)	frameshift	HEMI	**Path**	predicted null variant	Absent	Not reported	N/A	N/A	autoimmune hemolytic anemia and abnormal DNTCs	This study
60	18	M	African American	*MAGT1*	Immunodeficiency, X-linked, with magnesium defect, Epstein-Barr virus infection and neoplasia (XLR)	c.407G>A; p.(Trp136*)	nonsense	HEMI	Path	predicted null variant	Absent	Not reported	suspicious for ALPS	N/A	enlarged lymph nodes, cytopenias, anemia, thrombocytopenia, abnormal lymphocyte subsets, reported abnormal ALPS panel, and IgA nephropathy	This study
61	10	M	European American	*MAGT1*	Immunodeficiency, X-linked, with magnesium defect, Epstein-Barr virus infection and neoplasia (XLR)	c.223C>T; p.(Q75*)	nonsense	HEMI	Path	predicted null variant	Absent	Path(1), 539316	suspicious for ALPS (twice)	normal	N/A	[70, 71]
62	6	F	Unknown	*NRAS*	RAS-associated autoimmune leukoproliferative disorder (AD)	c.38G>A; p.(Gly13Asp)	missense	HET	Path	deleterious effect	All = 0.0004%; ASJ = 0.009%	Path(2)/LP(3), 13901	not consistent with a diagnosis of ALPS (X5)	decreased (first time); normal (1 year later)	N/A	[21, 72–75]

Reference sequences used (*ADA2*: NM_001282225.1; *CTLA4*: NM_005214.4; *KRAS*: NM_004985.5; *LRBA*: NM_006726.4; *MAGT1*: NM_032121.5; *NRAS*: NM_002524.5), Pt = Patient; M = Male; F = Female; HET = Heterozygous; HOMO = Homozygous; HEMI = Hemizygous; FH = Family history; Path = Pathogenic; LP = Likely Pathogenic; VUS = Variant of Unknown Significance (Bold means novel Variant); ALPS = Autoimmune Lymphoproliferative Syndrome. * Immunosuppressive therapy may affect the ALPS immunology test. Bold means novel variants

### Correlation of the ALPS Immune-panel Findings and Fas-mediated Apoptosis with Disease-causing Variants Detected in ALPS NGS Panel

**Fig. 2:**
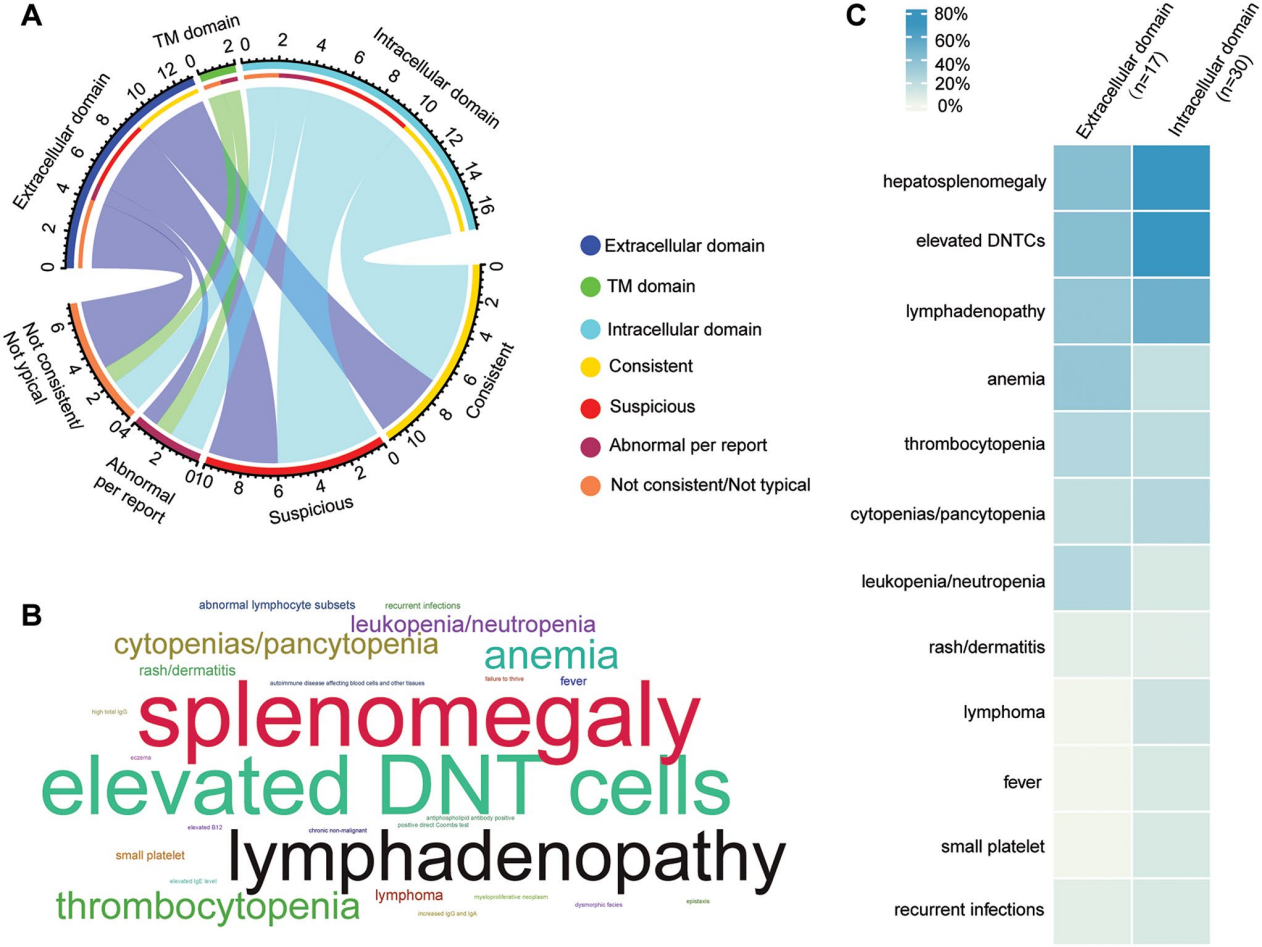
ALPS phenotypes and *FAS* genotypes correlation. (**A**) The correlation of ALPS immune-panel results with variants in different FAS protein domains. The numbers around the chord diagram represent the number of patients. (**B**) Cloud map shows the high frequency of patients’ phenotypes. Splenomegaly counts in 31 of a total 52 *FAS*-positive patients, elevated double negative T (DNT) cells counts in 28 of 52 (Including cases with the abnormal ALPS immune-panel results), and lymphadenopathy counts in 24 of 52 (Including enlarged lymph nodes and lymphadenopathy). (**C**) Heatmap showed the percentage of different phenotypes in *FAS*-positive patients, grouped by *FAS* variant’s location in terms of the extracellular and intracellular domains

Out of the 52 *FAS*-mutant cases, 32 cases had ALPS Immune-panel completed, either by our institution or elsewhere (per the provider report). We summarized the affected domains of *FAS* variants detected in these 32 cases and their ALPS Immune-panel results in Fig. 2A and Suppl. Table 2. Although not knowing whether the patients received immunosuppressive drugs at the time of testing, all but two of the 17 cases with pathogenic/likely pathogenic ICD variants had abnormal ALPS Immune-panel results, reporting “consistent with ALPS” or “suspicious for ALPS”. However, only 9/13 (69%) cases with ECD variants had an abnormal ALPS Immune-panel. This result is consistent with the reported incomplete penetrance of the ECD germline *FAS* pathogenic variants, especially those with a truncating effect [13, 16]. The four cases with ECD *FAS* variants and “not typical” or “not consistent” ALPS Immune-panel results all had frameshift variants, predicted to result in haploinsufficiency (Pts 2, 4, 6, 16 in Table 2).

Where available, we also evaluated the patients’ phenotypes and the phenotype-genotype correlation from the 52 *FAS*-positive cases. Splenomegaly, lymphadenopathy, and elevated DNTs were the top three phenotypes in these patients (Fig. 2B). Consistently, patients with ECD *FAS* pathogenic/likely pathogenic variants showed more reduced penetrance for the characteristic clinical phenotype of ALPS, compared to the ICD variants (Fig. 2C).

Of the 52 *FAS*-positive cases, ten cases underwent Fas-mediated apoptosis testing. One case failed due to technical issues, and 9/9 (100%) cases with a valid apoptosis test showed decreased Fas-mediated apoptosis (Table 2). This result is consistent with germline *FAS* pathogenic variants invariably having defective Fas-mediated apoptosis. This confirmed previous findings that the penetrance of abnormal in vitro Fas-mediated apoptosis in ALPS-FAS is approximately 100%, regardless of the variant location [13, 25].

Among the 10 cases positive for other ALPID, 8 cases had ALPS Immune-panel results (Table 3). While the majority of cases (6/8) showed “not consistent” or “not typical” results, two patients (Pts 60 and 61 in Table 3) showed “suspicious for ALPS” results, and both of them were hemizygous for pathogenic variants in *MGAT1*. These findings underscore the significance of genetic testing in facilitating the accurate diagnosis of ALPS.

### Correlation of the ALPS Immune-panel and Fas-mediated Apoptosis in Patients with VUS in *FAS*

Of the 66 cases who had a VUS in the ALPS NGS Panel, 31/66 (47%) cases had ALPS Immune-panel tests performed in our institution or elsewhere (per the provider report). Five cases had *FAS* VUS and “consistent” or “suspicious” results for ALPS Immune-panel (Table 4). Three of these cases had Fas-mediated apoptosis tests performed and found two were abnormal. Specifically, Pt 64 is heterozygous for a previously unreported synonymous variant, c.390A>G p.(Lys130=). This variant is predicted to enhance a cryptic splice donor site 4-bp upstream of this change. Per the provider’s report, this patient met the clinical criteria for ALPS, although the available information for this variant was not sufficient to classify it as likely pathogenic.

**Table 4:** *FAS* VUS in 802 patients with suspected ALPS and an abnormal ALPS Immune-panel result

Pt ID	Age (yrs)	Sex	Race/ ethnicity	Variant	Variant type	zygosity	Classification	In silico	gnomAD	ClinVar	FAS domain	ALPS Immune-panel	apoptosis	Family history and clinical indication	Ref
63	6	M	European American	**c.328G>A; p.(Gly110Arg)**	missense	HET	VUS	no impact	Absent	not reported	extracellular	consistent with ALPS; * not consistent with a diagnosis of ALPS (two years later)	normal (twice)	N/A	This study
64	18	F	European American	**c.390 A>G; p.(Lys130=)**	synonymous	HET	VUS	may enhance a nearby cryptic splice donor	Absent	not reported	extracellular	consistent with ALPS	decreased	increased IgA, IgG, Vit B12, sFASL with decreased apoptosis; splenomegaly; per report, met the diagnosis of ALPS	This study
65	6	M	European American	**c.676+5G>A; p.(?)**	splicing	HET	VUS	uncertain impact on nearby splice donor site	Absent	not reported	intracellular	consistent with ALPS	N/A	hepatosplenomegaly, enlarged lymph nodes, thrombocytopenia, abnormal ALPS panel and elevated B12	This study
66	14	M	European American	**c.710C>T; p.(Ala237Val)**	missense	HET	VUS	uncertain impact	All = 0.0004% (1 HET); SA = 0.003%	not reported	Intracellular (death domain)	consistent with ALPS	decreased (twice)	hepatosplenomegaly, jaundice, anemia, and enlargement of lymph nodes	This study
67	6	M	European American	**c.798T>A; p.(Asn266Lys)**	missense	HET	VUS	no impact	Absent	not reported	Intracellular (death domain)	suspicious for ALPS	N/A	hepatosplenomegaly	This study

*FAS* reference sequence: NM_000043.5, Age: age of testing; Pt = Patient; M = Male; F = Female; HET = Heterozygous; FH = Family history; Path = Pathogenic; LP = Likely Pathogenic; ALPS = Autoimmune Lymphoproliferative Syndrome; * Immunosuppressive therapy may affect the ALPS immunology test. Bold means novel variants

The other patient (Pt 66) is heterozygous for a missense variant located in the intracellular death domain, c.710C>T p.(Ala237Val). This variant has not been published. It has been reported in the gnomAD database in one heterozygote in all populations. REVEL score is 0.545 suggesting uncertain prediction on variant effect. Family studies showed his mother and sibling did not carry this variant, but his father was not available for testing.

### Performance of the ALPS NGS Panel in Patients with Suspected ALPS and Abnormal Immune-panel and/or Fas-mediated Apoptosis Results

Due to the lower (7.7%) diagnostic rate observed in our 802-patient cohort compared to the reported attribution of *FAS*, *FASLG*, and *CASP10* to ALPS [26], we systematically reviewed cases with the ALPS Immune-panel and/or the Fas-mediated apoptosis tests performed at our institution from November 2019 to January 2023. This was correlated with ALPS NGS sequencing results, to evaluate the diagnostic rate of ALPS NGS panel on patients with suspected ALPS and abnormal ALPS immunology results.

During the specified period, a total of 254 ALPS NGS panel cases were processed at our institution. 101/254 (40%) cases also underwent testing with the ALPS Immune-panel. 81/101 (80%) cases showed “inconsistent” or “not typical” results for ALPS. 20/101 (20%) were “suspicious for ALPS” or “consistent with ALPS”. In these twenty cases, 6 (30%) were positive for *FAS* pathogenic or likely pathogenic variants that supported the ALPS diagnosis (Suppl. Table 3), which was much higher than the diagnostic rate from the ALPS “immunology-unscreened” cohort.

Two patients were heterozygous for previously unreported VUS in *FAS* (Pts 63, 64). Of the remaining twelve patients with abnormal ALPS Immune-panel but no detected *FAS* variants, six underwent Fas-mediated apoptosis, revealing abnormal findings in two cases (Pts 70, 76 in Suppl. Table 3). These findings suggest that patients with suspected ALPS might be classified with ALPS-U or had pathogenic variants in the deep intronic regions/UTR regions in *FAS*,* FADD*,* FASLG*, or *CASP10*, which were not covered/detected in this genetic test. They could also have a somatic *FAS* variant with less than 5% VAF, or somatic *FADD*, *FASLG* or *CASP10* which was not targeted in this panel. Additional genetic testing might be warranted.

## Discussion

ALPS predominantly is caused by *FAS* mutations, either germline or somatic in DNT cells [15, 27]. In this study, we examined 802 patients with suspected ALPS referred to our institution for ALPS NGS panel from May 2014 to January 2023. The diagnosis yield of this ALPS NGS panel was 7.7%, which was lower than the reported attribution of *FAS*, *FASLG*, and *CASP10* to ALPS [26]. This suggests that the ALPS NGS panel may have been utilized more as a screening test for patients with suspected ALPS, or a lymphoproliferative and/or autoimmune disorder in general, which was recently proposed to be termed as ALPID [7].

Having more complete clinical information, particularly regarding the fulfillment of ALPS criteria could potentially increase the genetic diagnosis rate, as shown by the 20 cases, with abnormal ALPS immunology testing results, evaluated between November 2019 and January 2023. In this smaller cohort, the diagnostic yield of the ALPS NGS panel was much higher at 30% when including cases with abnormal ALPS immunology criteria.

**Fig. 3:**
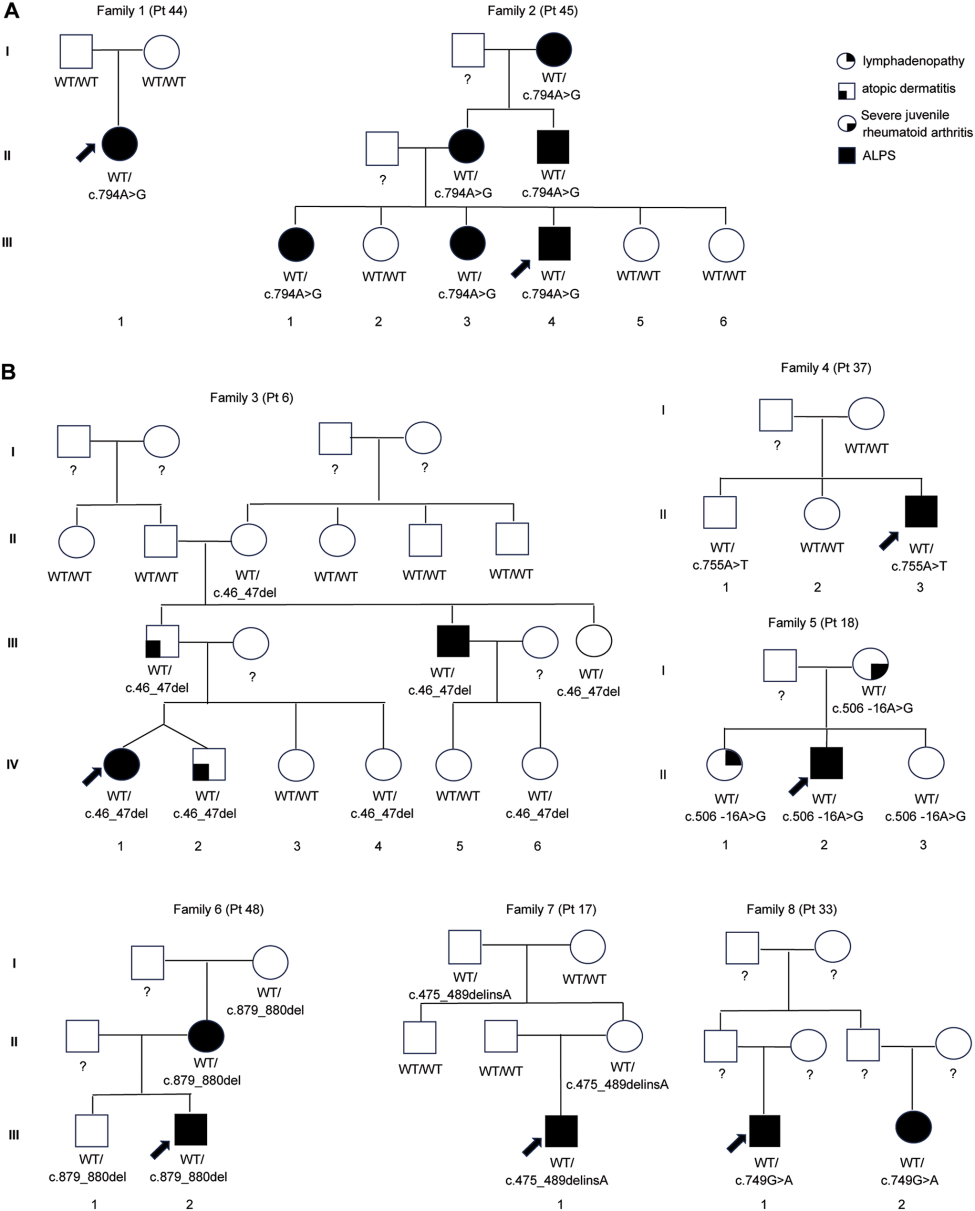
Family study results. (**A**) c.794A>G; p.(Asp265Gly), was detected as *de novo* in one family, and in the other family, this variant segregated with disease. (**B**) other representative family study results. The black arrows indicate the probands. Circle = female, square = male. The legend for the keys is at the top of the figure

The nine exons of the *FAS* gene encode a signal sequence in exons 1–2 that is cleaved upon trafficking of the Fas protein to the cell surface, three cysteine-rich ECDs in exons 3–5, a TM in exon 6, and ICDs spanning exons 7–9 comprising the exon 9-encoded death domain that interacts with the intracellular apoptosis-inducing signal transduction pathway [28]. We detected variants across all of these regions with the greatest number of pathogenic variants detected in the intracellular death domain (Fig. 1). The reduced penetrance of ALPS phenotypes of ECD variants has also been observed in our data (Fig. 1; Families 3 and 7 in Fig. 3B).

Variants in intron 5 and exon 6 are interesting and have been reported to result in skipping of exon 6 and producing a soluble Fas molecule that lacks the transmembrane domain [16, 29–34]. This soluble isoform blocks apoptosis induced by Fas. However, it has been proposed that haploinsufficiency, rather than the effect of an excessive production of soluble Fas, is the underlining disease mechanism [29]. This group of pathogenic variants is associated with a low penetrance of disease phenotype [29] like other variants in ECDs [16].

In our cohort, we detected three variants associated with exon 6 skipping in 5 probands, c.506-16A>G (Pt 18), c.568G>A which located at the last base of exon 6 (Pt 22), and c.506-3C>T (Pts 19, 20, 21). The c.506-16A>G and c.568G>A variants have previously been reported in patients with ALPS, both in a homozygous state [33] and in a heterozygous state, with cDNA sequencing showing in-frame skipping of exon 6 (p.Gly169_Trp189del). This results in a protein lacking the transmembrane domain [16].

Focusing on several illustrative cases; the c.506-16A>G variant was detected in a 15-year-old male with a clinical history of cardiac transplant, post-transplant lymphoproliferative disease (PTLD) with > 25% DNT cells, hepatosplenomegaly, enlarged lymph nodes, lymphoma, and sensorineural deafness (Pt 18, Table 2; Fig. 3B). Family studies showed this variant was inherited from his mother who had severe juvenile rheumatoid arthritis. His 17-year-old sister carries the variant and had left conductive hearing loss, atrial septal defect, heart palpitations, reduced exercise tolerance, and lymphadenopathy. His 12-year-old sister also carries this variant but without any relevant symptoms suggestive of ALPS. Family studies enabled the identification of at-risk family members and additional follow-up in this family.

The c.506-3C>T variant has not been previously reported in patients with ALPS, though a functional study using a minigene system showed it to lead to exon 6 skipping [30]. We detected c.506-3C>T in the heterozygous state in three male probands (Pts 19, 20, 21) with splenomegaly, red cell anemia, and thrombocytopenia as the common features. Patient 19 also had enlarged lymph nodes and reported abnormal ALPS Immune-panel. Patient 21 had neutropenia, intermittent rashes, and a paternal family history of ALPS. Though previously unreported in ALPS patients, c.506-3C>T is one of the top three common diagnostic variants detected in our cohort (Fig. 1B; Table 2).

It has been known that the most common genetic causes of ALPS are monoallelic pathogenic *FAS* variants followed by somatic *FAS* variants restricted to DNT cells [26]. Like ALPS-FADD, the combination of a germline *FAS* mutation and a somatic event (either mutations or LOH) impairing the second *FAS* allele (“2 hits”) has also been reported and accounts for the onset of clinical phenotype in ALPS, especially for ECD haploinsufficiency variants that have lower penetrance [35]. Detection of somatic *FAS* variants historically has been done by Sanger sequencing in sorted DNT cells. However, this procedure is expensive and technically challenging. NGS testing using DNA from whole blood has recently been reported to be able to detect somatic *FAS* mutations in seven ALPS patients [36] with *FAS* variant allele fraction ranging from 1.9 to 11.5% (median 5.9%). However, in our 254 cases from November 2019 to January 2023, in which the somatic *FAS* detection was included in the pipeline, we did not detect any somatic *FAS* variants. It is possible our somatic variant detection sensitivity (5%) is not high enough to detect these variants. For patients meeting ALPS criteria, with “consistent” or “suspicious” ALPS Immune-panel results, an abnormal Fas-mediated apoptosis, and a negative family history, further testing for somatic variants using sorted DNTC or NGS panel with higher sensitivity (< 5%) for somatic variants should be considered.

One limitation of the custom designed ALPS NGS panel is its lack of flexibility to add new genes to the panel. As additional genes associated with ALPID are identified, for example, *PIK3CD* [37, 38] and *PIK3R1* [39, 40], regularly updating the custom NGS panel would increase the diagnostic yield. If not seeking somatic *FAS* variants, physicians could also consider ordering WES-based or WGS-based testing for patients with suspected ALPS as the sequencing cost continues to decrease. For patients who underwent genetic testing before the NGS approach was adopted, it might be worthwhile to retest, while re-analysis of WES-based testing might also be considered.

However, the number of VUS detected and reported may pose challenges to its clinical utility. Family studies to see whether the variant is *de novo* in the proband or whether the variant segregates with the disease in the family will provide additional evidence to upgrade or downgrade VUS. For example, the c.794A>G p.(Asp265Gly) variant detected in two independent families (Pts 44 and 45) was originally reported as VUS in 2016. Subsequent family studies revealed that it was *de novo* in one family and segregated with disease in another family (Fig. 3A). This additional information allowed us to upgrade this VUS to likely pathogenic in our database before this variant was reported in the literature [41, 42].

Importantly, complete clinical phenotypes and biological correlates, such as those obtained through specific ALPS-related studies (ALPS Immune-panel, cytokines, Vitamin B12, and Fas-mediated apoptosis) would be helpful to provide supporting evidence for variant classification and should be considered in the variant classification work up. Utilizing additional markers, such as CD38, HLA-DR, and CD45RA [13, 43, 44], to discriminate between ALPS-specific DNTs and DNTs not related to ALPS would also be helpful in differentiating ALPS from other ALPID and increasing the genetic diagnostic yield of ALPS.

Our study, while reporting novel genetic findings from a large cohort of patients with suspected ALPS, has its limitations. It is a retrospective study conducted at a single center, potentially limiting its generalizability. Most cases were ordered from outside institutions, resulting in limited information regarding the patient’s clinical history (collected from the patient’s requisitions). Not all patients had ALPS immune results and even for cases with a result, we did not know whether they were on immunosuppressive drugs, which may influence the ALPS Immune-panel results and potentially somatic variant detection. These factors all affect the diagnostic rate.

In summary, we report the investigation of an NGS targeted gene panel on a large cohort of patients with suspected ALPS. Identification of *FAS* or other ALPID gene mutations with this NGS strategy is a cost- and time-effective option to expedite the diagnosis and treatment of these patients avoiding further complications due to a delayed diagnosis, although most cases remain genetically undiagnosed. Additional functional tests, including RNA sequencing, and family studies on VUS may further resolve uncertain results.

### Electronic Supplementary Material

Below is the link to the electronic supplementary material.

Supplementary Material 1

## Data Availability

Data is provided within the manuscript or supplementary information files.
