# Supplementary material for: Genetic Testing in Patients with Autoimmune Lymphoproliferative Syndrome: Experience of 802 Patients at Cincinnati Children’s Hospital Medical Center
Source: J Clin Immunol. 2024 Jul 26;44(7):166. doi: 10.1007/s10875-024-01772-z (PMC11282156; doi:10.1007/s10875-024-01772-z)
Supplement: Supplementary file 1 — Supplementary Material 1 [file 10875_2024_1772_MOESM1_ESM.docx]

Supplementary material:

DNA Extraction and NGS test

Genomic DNA was extracted using the Chemagic™ Magnetic Separation Module I instrument (PerkinElmer, Waltham, MA) or manually using Qiagen Puregene kit (Qiagen, Germany). The coding regions, flanking 20 bp intronic and untranslated regions (5’ and 3’) of 9 genes (*CASP8*, *CASP10*, *FADD*, *FAS*, *FASLG*, *ITK*, *KRAS*, *MAGT1*, *NRAS*) were enriched/captured using microdroplet PCR technology (RainDance Technologies Inc.) for 548 cases from May 2014 to October 2019. For 254 cases analyzed from November 2019 to January 2023, the coding regions and their flanking 20 bp regions, as well as select pathogenic variants (in the Human Gene Mutation Database version 2018.4) in the promoter and deep intronic regions of 15 genes were enriched/captured using the SureSelect System (Agilent Technologies Inc.). This design included 6 additional genes: *ADA2 (CECR1)*, *CTLA4*, *LRBA*, *PRKCD*, *RASGRP1*, and *STAT3*. The enriched targets were sequenced on the Illumina instrument (Illumina Inc., USA) with either >=20-fold coverage (for cases before October 2019) or >= 50-fold coverage (for cases after November 2019) at every target base. Variants were classified according to the ACMG guidelines[7]. Somatic variants with >=5% allele fraction in *FAS* were targeted after November 2019. Regions without enough coverage, variant confirmation, and family studies, were analyzed by Sanger sequencing. Larger deletions, insertions and recombinational events were not detected using this method. Each ALPS NGS panel was reviewed with input from both a clinical immunologist and a clinical molecular geneticist.


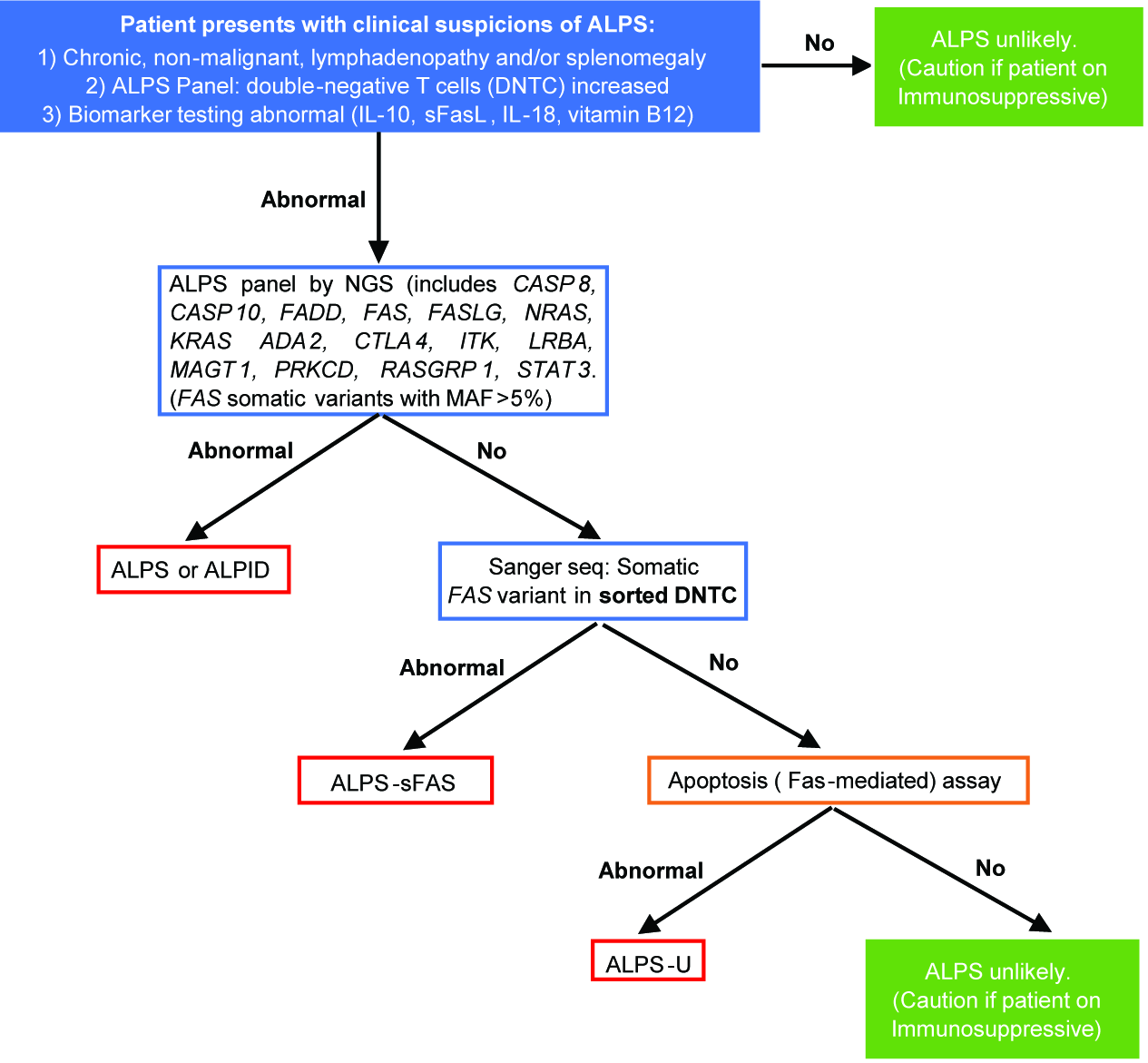


Supplemental Figure 1. ALPS diagnostic testing algorithm

Supplemental Table 1. Uncertain diagnosis in cases with a heterozygous Path/LP variant in genes associated with recessive conditions.

| **Pt ID** | **Age (Yrs)** | **Sex** | **Race/ ethnicity** | **Gene** | **Variant** | **Variant type** | **Zygosity** | **In silico prediction** | **Classification** | **Diagnostic** | **gnomAD** | **ClinVar** | **OMIM** | **ALPS Immune-panel** | **Fas-mediated apoptosis** | **Family history and clinical indication** | **Ref** |
| --- | --- | --- | --- | --- | --- | --- | --- | --- | --- | --- | --- | --- | --- | --- | --- | --- | --- |
| 80 | 16 | F | Latino-Hispanic | *ADA2* | c.660C>G; p.(Tyr220*) | non-sense | HET | predicted null variant | Path | uncertain | All: 0.0004 %   AMR: 0.003 % | not reported | Vasculitis, autoinflammation, immunodeficiency, and hematologic defects syndrome (AR). | N/A | N/A | N/A | [1] |
| 81 | 6 | M | Latino-Hispanic/Venezuela | *LRBA* | **c.2166-1G>C; p.(?)** | splicing | HET | predicted null variant | LP | uncertain | Absent | LB(1), 1066609 | Immunodeficiency, common variable, 8, with autoimmunity (AR) | N/A | N/A | N/A | This study |
| 82 | 3 | M | Latino-Hispanic | *LRBA* | c.1931dup; p.(Arg645Alafs*3) | frame-shift | HET | predicted null variant | Path | uncertain | All: 0.002%; AMR: 0.1% | PA(1), 863114 | Immunodeficiency, common variable, 8, with autoimmunity (AR) | *not consistent with a diagnosis of ALPS | N/A | N/A | [2, 3] |

Reference sequences used (*ADA2*: NM_001282225.1; *LRBA*: NM_006726.4). Pt=Patient; M=Male; F=Female; HET=Heterozygous; HOMO=Homozygous; HEMI=Hemizygous; FH=Family history; Path=Pathogenic; LP=Likely Pathogenic; VUS=Variant of Unknown Significance (Bold means novel Variant); ALPS= Autoimmune Lymphoproliferative Syndrome. * Immunosuppressive therapy may affect the ALPS immunology test. Bold means novel variants.

Supplemental Table 2. The correlation of location domains of the Path/LP variants in *FAS* and ALPS Immune-panel results

|  | abnormal ALPS Immune-panel | | | ALPS Immune-panel: not typical/not consistent with ALPS |
| --- | --- | --- | --- | --- |
|  | consistent | suspicious | per report |  |
| Extracellular domain | 4 (31%) | 4(31%) | 1(7%) | 4(31%) |
| TM domain | 0 | 0 | 1(50%) | 1(50%) |
| Intracellular domain | 7(41%) | 6(35%) | 2(12%)* | 2(12%) |

Path/LP=pathogenic/likely pathogenic; TM=transmembrane

*Patient 42 had both a provider-reported abnormal ALPS Immune-panel result and a “not consistent with ALPS” in-house ALPS Immune-panel result. Pt 42 is heterozygous for a previously reported pathogenic *FAS* variant in the intracellular death domain [18, 47]. The ALPS Immune-panel discrepancy seen here in Pt 42 might be due to immunosuppressive therapy already in use which may affect the ALPS immunology test done in our lab.

Supplemental Table 3. The ALPS NGS results in 20 patients with suspected ALPS and an abnormal ALPS Immune-panel result.

| **Pt ID** | **ALPS Immune-panel** | **Fas-mediated Apoptosis** | **NGS report** | **(Gene)Variant** | **Family history and clinical indication** |
| --- | --- | --- | --- | --- | --- |
| 7 | Consistent with ALPS | N/A | positive | **NM_000043.5(FAS):c.146T>A; p.(Leu49*) het, LP** | N/A |
| 9 | Suspicious for ALPS; *Not typical for a diagnosis of ALPS (6 months later) | N/A | positive | **NM_000043.5(FAS):c.259G>T; p.(Glu87*) het, Path** | N/A |
| 11 | Consistent with ALPS | N/A | positive | NM_000043.5(FAS):c.332A>G; p.(His111Arg) het, Path | enlarged lymph nodes, red cell anemia, reported abnormal ALPS panel, increased IgG and IgA |
| 13 | Suspicious for ALPS | N/A | positive | NM_000043.5(FAS):c.334+3A>C; p.(?) het, LP | N/A |
| 29 | Consistent with ALPS | N/A | positive | **NM_000043.5(FAS):c.730C>T; p.(Gln244*) het, Path** | N/A |
| 31 | Suspicious for ALPS | Decreased | positive | NM_000043.5(FAS):c.748C>T; p.(Arg250*) het, Path | hepatosplenomegaly, reported abnormal ALPS panel, thrombocytopenia, family history of lymphoma and myeloproliferative neoplasm |
| 63 | Consistent with ALPS; *Not consistent with a diagnosis of ALPS (2 years later) | Normal X2 | uncertain | **NM_000043.5(FAS):c.328G>A; p.(Gly110Arg) het, VUS** | N/A |
| 64 | Consistent with ALPS; *Not typical for a diagnosis of ALPS (4 months later) | N/A | uncertain | **NM_000043.5(FAS):c.390A>G; p.(Lys130=) het, VUS** | increased IgA, IgG, Vit B12, sFASL with decreased apoptosis; splenomegaly; per report, met the diagnosis of ALPS |
| 68 | Suspicious for ALPS | Normal | uncertain | **NM_006726.4(LRBA):c.2164C>T; p.(Arg722Cys) het, VUS** | ITP, autoimmune hemolytic anemia and autoimmune neutropenia |
| 69 | Suspicious for ALPS | N/A | uncertain | **NM_006726.4(LRBA):c.4694A>G; p.(Glu1565Gly) het, VUS** | History of renal failure s/p renal transplantation, VATER syndrome, cytopenias, flow cytometry confirming for ALPS |
| 70 | Consistent with ALPS | Decreased | negative | N/A | N/A |
| 71 | Consistent with ALPS | N/A | negative | N/A | A year-long history of splenomegaly and pancytopenia complicated by a recent likely autoimmune-mediated thrombocytopenia, now with a positive ALPS screen |
| 72 | Suspicious for ALPS | Normal | negative | N/A | recurrent lymph node swelling |
| 73 | Suspicious for ALPS | N/A | negative | N/A | fever(s), failure to thrive, (hepato)splenomegaly, lethargy, enlarged lymph nodes, cytopenia, anemia, abnormal ALPS panel |
| 74 | Suspicious for ALPS | N/A | negative | N/A | N/A |
| 75 | Suspicious for ALPS | N/A | negative | N/A | thrombocytopenia/small platelets, chronic ITP, lymphoma |
| 76 | Suspicious for ALPS | Slightly decreased | negative | N/A | N/A |
| 77 | Suspicious for ALPS; *Not consistent with a diagnosis of ALPS (2 years later) | Normal | negative | N/A | N/A |
| 78 | Suspicious for ALPS; *Not consistent with a diagnosis of ALPS (2 months later) | Normal X2 | negative | N/A | cytopenias, ITP, hemolytic anemia, autoimmune neutropenia, abnormal ALPS panel, low or absent NK function |
| 79 | Although the assay results are not diagnostic for ALPS, the patterns are suggestive and consistent with ALPS; suspicious for ALPS (two months later) | N/A | negative | N/A | enlarged lymph nodes, leukopenia/neutropenia, thrombocytopenia/small platelets, abnormal ALPS panel |

ALPS= Autoimmune Lymphoproliferative Syndrome; FH=Family history; HET=Heterozygous; HOMO=Homozygous; HEMI=Hemizygous; ITP = Immune Thrombocytopenic Purpura; Pt=Patient; M=Male; F=Female; Path=Pathogenic; LP=Likely Pathogenic; VUS=Variant of Unknown Significance (Bold means novel Variant). * Immunosuppressive therapy may affect the ALPS immunology test. Bold means novel variants.

1. Hashem, H., et al., *Hematopoietic Cell Transplantation Cures Adenosine Deaminase 2 Deficiency: Report on 30 Patients.* J Clin Immunol, 2021. **41**(7): p. 1633-1647.

2. Fernandez, K.S., R. Antony, and A. Kumar, *Patients with "ALPS-like phenotype" diagnosed with immune dysregulation due to LRBA deficiency.* Pediatr Blood Cancer, 2019. **66**(3): p. e27558.

3. Tang, W.J., et al., *Potential protein-phenotype correlation in three lipopolysaccharide-responsive beige-like anchor protein-deficient patients.* World J Clin Cases, 2021. **9**(21): p. 5873-5888.
